# Clinical Significance of Heparanase Splice Variant (T5) in Renal Cell Carcinoma: Evaluation by a Novel T5-Specific Monoclonal Antibody

**DOI:** 10.1371/journal.pone.0051494

**Published:** 2012-12-12

**Authors:** Uri Barash, Gil Arvatz, Roy Farfara, Inna Naroditsky, Ilana Doweck, Sari Feld, Ofer Ben-Izhak, Neta Ilan, Ofer Nativ, Israel Vlodavsky

**Affiliations:** 1 Cancer and Vascular Biology Research Center, Rappaport Faculty of Medicine, Technion, Haifa, Israel; 2 Department of Urology, Bnai-Zion Medical Center, Haifa, Israel; 3 Department of Pathology, Rambam Health Care Campus, Haifa, Israel; 4 Department of Otolaryngology, Head and Neck Surgery, Carmel Medical Center, Haifa, Israel; Fox Chase Cancer Center, United States of America

## Abstract

T5 is a novel splice variant of heparanase, an endo-β-D-glucuronidase capable of cleaving heparan sulfate side chains at a limited number of sites. T5 splice variant is endowed with pro-tumorigenic properties, enhancing cell proliferation, anchorage independent growth and tumor xenograft development despite lack of heparan sulfate-degrading activity typical of heparanase. T5 is over expressed in the majority of human renal cell carcinoma biopsies examined, suggesting that this splice variant is clinically relevant. T5 is thought to assume a distinct three-dimensional conformation compared with the wild type heparanase protein. We sought to exploit this presumed feature by generating monoclonal antibodies that will recognize the unique structure of T5 without, or with minimal recognition of heparanase, thus enabling more accurate assessment of the clinical relevance of T5. We provide evidence that such a monoclonal antibody, 9c9, preferentially recognizes T5 compared with heparanase by ELISA, immunoblotting and immunohistochemistry. In order to uncover the clinical significance of T5, a cohort of renal cell carcinoma specimens was subjected to immunostaining applying the 9c9 antibody. Notably, T5 staining intensity was significantly associated with tumor size (p = 0.004) and tumor grade (p = 0.02). Our results suggest that T5 is a functional, pro-tumorigenic entity.

## Introduction

Heparanase is an endo-β-glucuronidase that cleaves heparan sulfate (HS) side chains of heparan sulfate proteoglycans (HSPGs) presumably at sites of low sulfation, releasing saccharide products with appreciable size (4–7 kDa) that can still associate with protein ligands and facilitate their biological potency [1]–[3]. Mammalian cells express primarily a single dominant functional heparanase enzyme (heparanase-1). A second heparanase (heparanase-2) gene has been cloned based on sequence homology but apparently lacks HS degrading activity [4], [5]. Enzymatic degradation of HS leads to disassembly of the extracellular matrix (ECM) underlying endothelial and epithelial cells and is therefore involved in fundamental biological phenomena associated with tissue remodeling and cell migration, including inflammation, angiogenesis and metastasis [1], [2], [6], [7]. While a decisive role of heparanase in cellular invasion and tumor metastasis is well documented [1], [2], [7], [8], the function that heparanase plays in primary tumor progression is largely unknown, but likely involves angiogenic and signaling aspects [9]–[13].

Alternative splicing increases the coding capacity of the genome, generating multiple proteins from a single gene. The resulting protein isoforms frequently exhibit different biological properties that may play an essential role in tumorigenesis [14]–[16]. We have recently reported the identification and characterization of a novel spliced form of human heparanase, termed T5 [17]. In this splice variant, 144 bp of intron 5 are joined with exon 4, resulting in a truncated, enzymatically inactive protein. T5 splice variant is endowed with pro-tumorigenic properties, enhancing cell proliferation, anchorage independent growth and tumor xenograft development [17]. These features were observed in several tumor-derived cell lines over expressing T5, while T5 gene silencing was associated with reduced cell proliferation, suggesting that its function is relevant to multiple tumor types [17]. Notably, T5 mRNA expression is up-regulated in 75% of human renal cell carcinoma (RCC) biopsies examined, implying that this splice variant is clinically relevant [17]. T5 is thought to assume a distinct three-dimensional conformation compared with the wild type (*wt*) heparanase protein. We sought to exploit this presumed feature by generating monoclonal antibodies that will recognize the unique structure of T5 without, or with minimal recognition of heparanase, thus enabling more accurate assessment of the clinical relevance of T5. Here, we provide evidence that such a monoclonal antibody (mAb), 9c9, preferentially recognizes T5 compared with heparanase by ELISA, immunoblotting and immunohistochemistry. A cohort of renal cell carcinoma specimens was subjected to immunostaining applying mAb 9c9. Notably, T5 staining intensity was significantly associated with tumor size (p = 0.004) and tumor grade (p = 0.02), suggesting that T5 is a functional, pro-tumorigenic entity.

## Materials and Methods

### Cloning and Purification of Maltose Binding Protein (MBP)-T5

T5 was amplified by PCR using the following primers: forward 5' GGAATTCATGCTG CTGCGCTCG 3′ and reverse 5′AACTGCAGTCATTTCTTACTTGAGTAGG 3' and was inserted into bacterial expression vector (pMal-c2; NEB). The expression and purification of MBP and MBP-T5 was carried out according to the manufacture's (NEB) instructions. Briefly, MC1061 bacteria culture was grown in the presence of isopropyl-β-D-thiogalactopyranoside (IPTG; 0.07 mM) for 5 h at 16°C. Bacteria were then harvested by centrifugation (5,000 g; 10 min at 4°C); the pellet was re-suspended in column buffer [80 mM Na_2_PO_4_, 20 mM NaH_2_PO_4_ pH 7.5, 100 mM NaCl, 20 mM 4-(2-Aminoethyl) benzenesulfonyl fluoride hydrochloride (AEBSF)] and incubated with lysosyme (1 mg/ml; Sigma) for 30 min on ice. Following sonication and centrifugation, (20,000 g*;* 30 min), the supernatant was mixed gently with 1/10 volume of amylose resin (20 h; 4°C). The resin was then packed in a column, washed, and MBP-T5 was eluted with column buffer containing 10 mM maltose. Elution fractions were analyzed by SDS-PAGE and protein concentration was determined by the Bradford assay (Bio-Rad).

### Generation of Anti Human T5 Monoclonal Antibodies

BALB/c mice were immunized with 50 µg of MBP-T5 fusion protein in complete Freund's adjuvant (CFA; Sigma), followed by five injections of MBP-T5 (50 µg) in incomplete Freund's adjuvant (IFA) every 2 weeks. Splenocytes were then isolated, fused with NSO myeloma cells and hybridomas were screened for their ability to bind MBP-T5 or heparanase but not MBP by ELISA, essentially as described [4], [18], [19]. Positive hybridomas were selected, expanded and cloned. Hybridoma subclass was determined by isotyping kit according to the manufacturer's (Serotec, Oxford, UK) instructions. Monoclonal antibodies were purified from the cell supernatant by protein-G chromatography.

### Cell lysates and Protein Blotting

Preparation of cell lysates and immunoblotting analyses were carried out as described previously [17].

### Patients

The study included 66 patients with renal cancer (Table 1) that were diagnosed in the Department of Urology, Bnai-Zion Medical Center, Haifa, Israel, whose archival paraffin-embedded pathological material was available for immunohistochmical analysis. The study protocol was approved by the Bnai-Zion Medical Center Helsinki Committee Institutional Review Board (IRB). Being a retrospective study that include data retrieval from medical records and paraffin blocks, the local IRB do not require individual patients approval by signing a written inform consent. It should be mentioned that some patients have died during follow up. All patients underwent surgical removal of the renal tumor (radical nephrectomy or partial nephrectomy). The follow-up protocol included physical examination, imaging studies of the chest and abdomen, renal function tests, and urine analysis. Patients were seen every 4 months during the first 2 years after surgery; every 6 months 2–5 years postoperatively and yearly thereafter. The clinical and pathological data of all patients was reviewed and patients were re-staged according to the 2009 Americam Joint Committee on Cancer (AJCC) revision of the tumor-node-metastasis (TNM) staging system [20]. The following information was recorded: demographics, site of tumor, histological subtype, Fuhrman's nuclear grade, TNM stage, treatment modality, and status at the end of the study.

**Table 1:** Demographic and clinical description of patients.

Parameter	No of patients (%)
*Gender*	
Male	43 (65)
Female	23 (35)
*Grade* *	
1	7 (12)
2	21 (35)
3	20 (33)
4	12 (20)
*Histology* *	
Clear cell	45 (75)
papillary	5 (8)
sarcomatoid	7 (12)
other	3 (5)
*Tumor size* *	
<4	24 (40)
4–7	18 (30)
>7	18 (30)

* Data on 6 patients was missing.

### Immunostaining

Staining of formalin-fixed, paraffin-embedded 5 micron sections was performed essentially as described [17], [21], [22]. Briefly, slides were deparaffinized, rehydrated, and subjected to antigen retrieval by boiling (20 min) in 10 mM citrate buffer, pH 6.0. Following washes with phosphate buffered saline (PBS), slides were incubated with 10% normal goat serum (NGS) in PBS for 60 min to block non specific binding and incubated (20 h, 4°C) with mAb 9c9, diluted 1∶100 in blocking solution. Slides were extensively washed with PBS containing 0.01% Triton X-100 and incubated with a secondary reagent (Envision G/2 system/AP) according to the manufacturer's (Dako, Glostrup, Denmark) instructions. Following additional washes, color was developed with the permanent red reagent (Dako), sections were counterstained with hematoxylin and mounted, as described [21], [23]. Immunostained specimens were examined by senior pathologist (IN) who was blind to clinical data of the patients. Staining was scored according to the intensity of staining (0: none, +1: weak-moderate; +2: strong), and the percentage (extent staining) of tumor cells that were stained. The extent of staining was further categorized as low (0: <10%), moderate (+1∶10–50%) and high (+2>50% of the cells). Specimens that were similarly stained with mouse IgG, or applying the above procedure but lacking the primary antibody, yielded no detectable staining.

Immunofluorescent double staining of heparanase and T5 applying antibody #733 and mAb 9c9 was carried out essentially as described [21].

### Statistical Analysis

Univariate association between T5 staining intensity and clinical and pathological findings as well as patients outcome, were analyzed using Chi Square tests (Pearson, Fisher exact test).

## Results

### Generation and Characterization of T5-specific mAb

**Figure 1:**
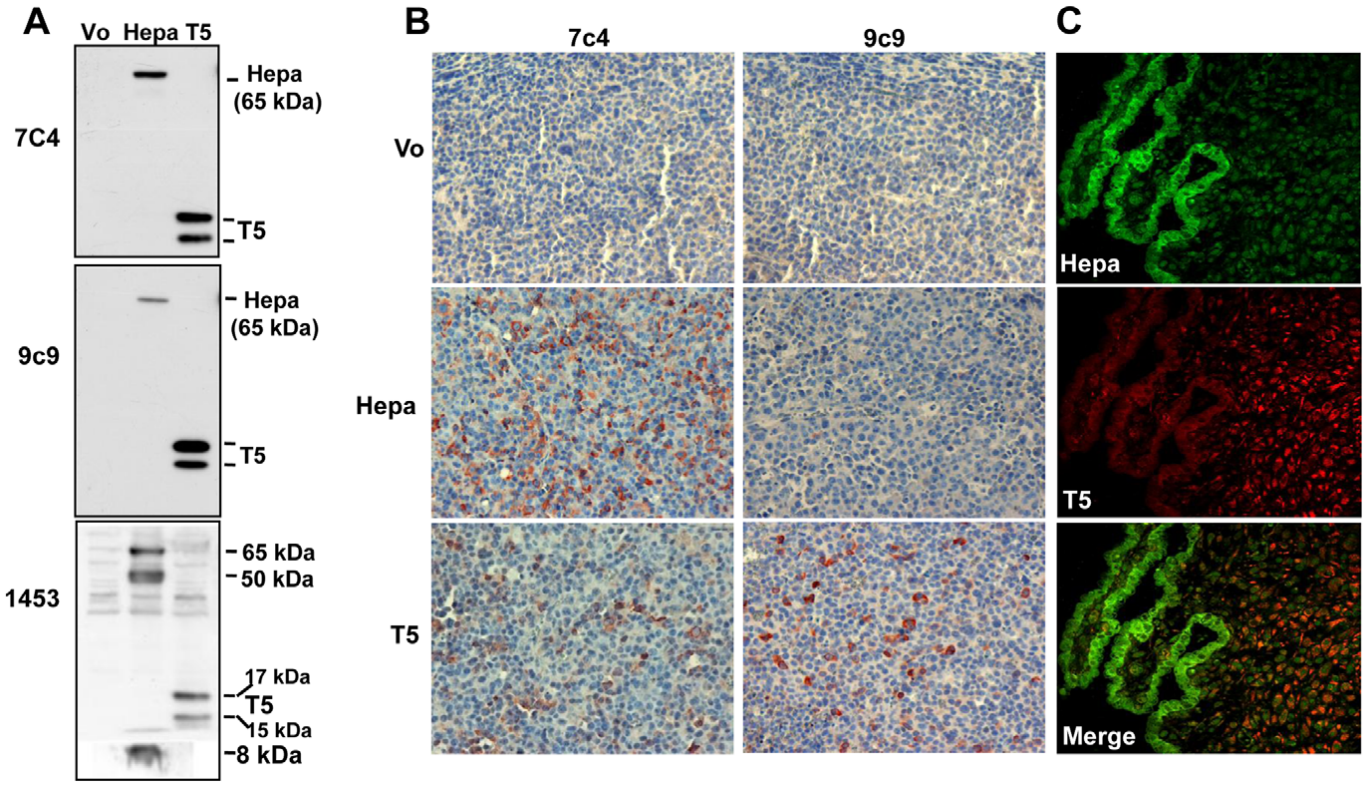
Characterization of anti T5 mAb. **A**. Immunoblotting. HEK 293 cells were transfected with heparanase (Hepa) or T5 gene constructs and lysate samples were subjected to immunoblotting applying mAb 7c4 (upper panel), mAb 9c9 (middle panel) or pAb 1453 (lower panel). Cells transfected with an empty vector (Vo) were used as control. Note that mAb 9c9 preferentially recognizes T5 vs. heparanase. **B**. Immunohistochemistry. Five micron sections of tumor xenografts produced by control CAG myeloma cells (Vo) or CAG cells over expressing heparanase (Hepa) or T5 were subjected to immunostaining applying mAb 9c9 as described under 'Materials and Methods'. Note that mAb 9c9 only reacts on sections derived from CAG cells over expressing T5. **C**. Human placenta. Placenta specimens were subjected to double immunofluorescent staining applying rabbit (pAb 733, green) and mouse (mAb 9c9, red) anti-heparanase antibodies. Merged image is shown in the lower panel. Note distinct expression pattern of heparanase (cytotrophoblasts) and T5 (villus stromal cells).

We have recently reported the cloning of T5, a functional splice variant of heparanase endowed with pro-tumorigenic properties [17]. In order to advance T5 research we developed a panel of monoclonal antibodies (mAb) against bacterially-expressed MBP-T5 fusion protein and compared their capacity to recognize T5 vs. heparanase by ELISA (Table S1). We have noticed that some mAbs recognize heparanase and T5 to a similar extent (i.e., 7c4) while others (i.e., 9c9) preferentially recognized T5 vs. heparanase (Fig. S1). Likewise, while antibody 7c4 recognized the latent 65 kDa heparanase and T5 to a similar extent by immunoblotting, antibody 9c9 preferentially reacts with T5 (15 and 17 kDa protein bands representing unglycosylated and glycosylated T5, respectively [17]; Fig. 1A). Notably, mAbs 7c4 and 9c9 did not recognize the 8 kDa and 50 kDa heparanase subunits (Fig. 1A), nor the single chain constitutively-active enzyme (GS3; not shown), suggesting that the epitope of both antibodies is localized in the linker region of heparanase which is removed by proteolytic processing and is not present in the 8 and 50 kDa subunits or the GS3 variant [9], [24] (Fig. S2A). These results suggest that the generation of T5-specific monoclonal antibodies is feasible.

In order to reveal whether our antibodies are suitable for immunohistochemical analysis, and to explore their specificity, tumor xenografts were subjected to immunostaining. Remarkably, the specificity observed by ELISA and immunoblotting was also seen in immunostaining. Thus, antibody 7c4 reacted similarly with tumor xenografts generated by CAG human myeloma cells over-expressing heparanase or T5 (Fig. 1B, left). In contrast, antibody 9c9 only reacted with tumor xenografts produced by CAG cells over-expressing T5 (Fig. 1B, right lower panel). Encouraged by these results, we next examined the performance of antibody 9c9 on human specimens. We first employed placenta tissue which exhibits high levels of heparanase expression [25], [26]. Heparanase staining was most intense in cytotrophoblasts (Fig. 1C, green), in agreement with previous results [26], [27]. Importantly, mAb 9c9 did not stain the cytotrophoblasts but rather the villus stromal cells (Fig. 1C, red). This result clearly demonstrates the specificity of mAb 9c9 staining towards T5 vs. heparanase and illustrates the tight control of heparanase mRNA alternative splicing, resulting in the generation of T5 in a discrete, cell-specific manner.

**Figure 2:**
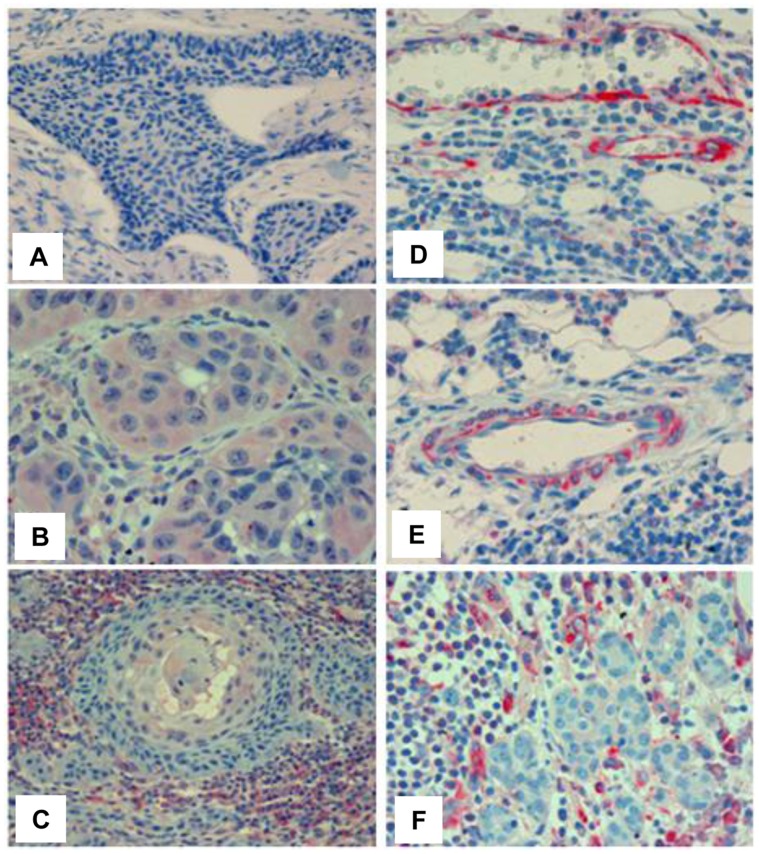
T5 staining in head and neck tumor biopsies. Tumor specimens were subjected to immunohistochemical analyses applying mAb 9c9 as described under 'Materials and Methods'. Shown are representative images of head and neck T5 negative (**A**) and positive tumors expressing T5 in tumor cells (**B**), tumor microenvironment (**C, F**), and tumor-associated blood vessels (**D, E**).

**Figure 3:**
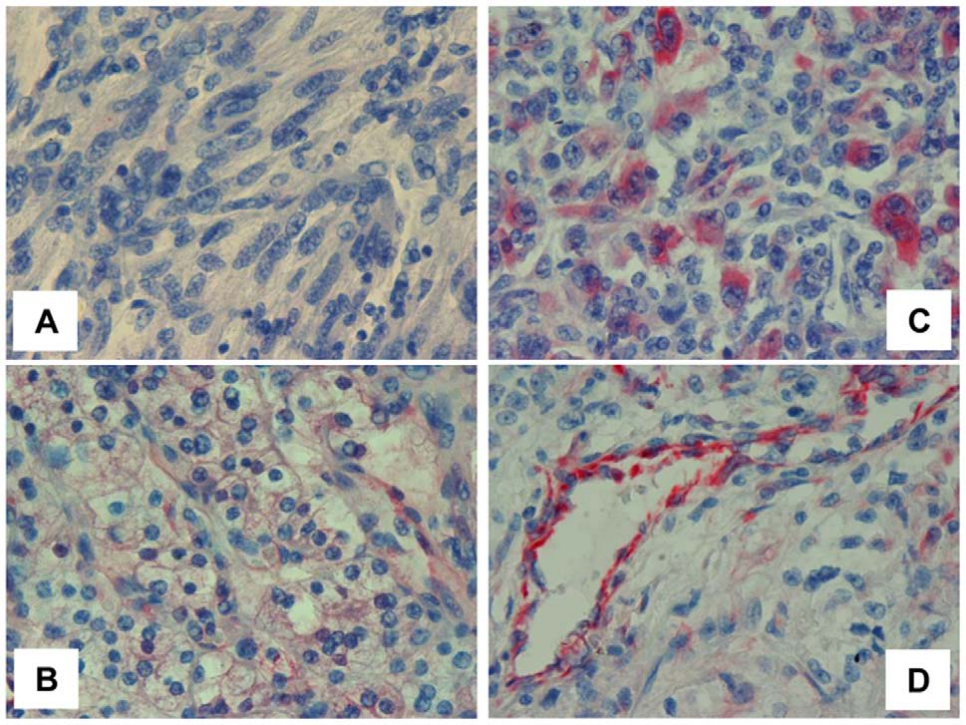
T5 staining in RCC. Tumor specimens were subjected to immunohistochemical analyses applying mAb 9c9 as described above. Shown are representative images of RCC T5 negative (**A**) and positive specimens of low grade clear cell carcinoma (**B**) and high grade sarcomatoid tumors (**C**). Staining of tumor-associated blood vessels is shown in (**D**).

We have next examined the capability of mAb 9c9 to detect T5 in tumor biopsies. In head and neck cancer, mAb 9c9 stained positively the epithelial compartment of some carcinomas (Fig. 2B); in other specimens, cells of the tumor microenvironment, predominantly mononuclear cells (Fig. 2F), rather than carcinoma cells stained positively for mAb 9c9 (Fig. 2C). In addition, mAb 9c9 nicely decorated endothelial cells lining blood vessels and adjacent pericytes in the tumor vasculature (Figs. 2D, E; 3D), in agreement with pro-angiogenic properties of T5 [17]. T5 staining at higher frequency and intensity was noted in renal cell carcinoma (Fig. 3).

### Clinical Relevance of T5 for RCC

We employed mAb 9c9 to examine T5 expression in specimens collected from RCC patients. Sixty six patients (43 male; 23 female) were included in the study (median age at diagnosis was 60.3±13.3), predominantly classified as clear cell carcinoma (75%). Demographic and clinical description of patients is shown in Table 1. T5 staining was observed in 67% of the cases (44/66; Fig. 3B, C), close in magnitude to T5 expression in RCC evaluated by RT-PCR (75%; [17]), whereas 33% (22/66) of the specimens were negative (Fig. 3A). T5 staining was associated with tumor grade (Table 2). Here, 32% of the cases scored negative for T5 were categorized as advanced grade (3+4) compared with 63% of the cases with high grade tumors that stained positively for T5 (p = 0.02). Notably, T5 staining was associated with tumor size (Table 3). Thus, 32% of the cases that were scored negative for T5 had tumors larger than 4 cm; in contrast, 73% of the cases that were stained positive for T5 had large tumors (Table 3), differences that are statistically highly significant (p = 0.004). There was no association between T5 and histological subtype, stage at presentation, or patients’ outcome.

**Table 2:** T5 staining associates with tumor grade in RCC.

	Tumor grade		
T5	1+2 (%)	3+4 (%)	Total
Negative	13 (68)	6 (32)	19
Positive	15 (37)	26 (63)	41
	28	32	60

P = 0.02.

* Data on 6 patients was missing.

**Table 3:** T5 staining associates with tumor size in RCC.

	Tumor size		
T5	<4 cm(%)	>4 cm(%)	Total
Negative	13 (68)	6 (32)	19
Positive	11 (27)	30 (73)	41
	24	36	60*

P = 0.004.

* Data on 6 patients was missing.

## Discussion

Alternative splicing considerably expands the information content of the transcriptome and proteome through the expression of different mRNAs from a single gene. Splicing abnormalities are a common characteristic of cancer, including genes associated with cell growth, motility, apoptosis, and response to chemotherapy [28], [29]. Alternative splicing closely affects also the tumor microenvironment. For example, alternative splicing of vascular endothelial growth factor (VEGF) generates protein variants capable or incapable of interacting with HS, exerting diverse angiogenic properties [30]. Similarly, HSPG (i.e., CD44) and other constituents of the ECM (i.e., fibronectin) are alternatively spliced, decisively mediating cell proliferation and tumor metastasis [31], [32]. Splice variants of xenopus, mole rat (Spalax), and human heparanase have been described [33]–[36], commonly lacking HS-degrading activity typical of heparanase and exerting as yet uncharacterized biological function.

We have recently described a human heparanase splice variant termed T5 [17]. T5 is composed of the 8 kDa subunit and linker segment of heparanase but lacks the 50 kDa subunit which contains the substrate (HS)-binding domains and catalytic residues (Fig. S2A). T5 is thus incapable of cleaving HS, yet endowed with pro-tumorigenic features thought to be mediated by Src activation [17]. Here, we expand the notion that T5 expression is associated with tumor progression. T5 expression was enhanced in most (67%) RCC specimens, correlating with larger tumors and higher histological grade (Tables 2, 3), two parameters that closely associate with patients' outcome [37], [38], thus providing a clinical relevance for T5. A key reagent that enabled the clinical evaluation of T5 is mAb 9c9. This unique antibody preferentially recognizes T5 vs. heparanase by ELISA, immunoblotting, and immunostaining (Fig. 1; Table S1). Immunohistochemical analysis applying mAb 9c9 is therefore expected to predominantly detect T5. It should be noted that unlike heparanase, T5 is not subjected to proteolytic processing [17]. Thus, lack of reactivity cannot be attributed to the loss of mAb 9c9 epitope but rather directly reflects T5 expression levels. Notably, distinct clinical associations were found for heparanase and T5. Hence, while heparanase staining in RCC was associated with histological subtype, distant metastasis, and patients' survival [39], [40], T5 staining was not associated with these parameters but rather with tumor size and tumor grade (Tables 2, 3). This suggests that heparanase and T5 affect different aspects of RCC tumor progression which complement each other. Interestingly, mAb 9c9 as well as mAbs 7c4, 2G7 and 5F8 did not recognize the 8 or 50 kDa heparanase subunits and their epitope was therefore concluded to be localized to the linker segment (Table S1). In contrast, mAbs 9D5 and 5B5 recognized the 8 kDa subunit, and further deletion mutagenesis analyses localized their epitope to the protein N-terminus (Table S1; Fig. S2B). This is in agreement with the three-dimensional structure predicted for heparanase in which the protein N-terminus appears unstructured, exposed, and likely immunogenic [41]. The model, however, was predicted for the constitutively-active variant (GS3) lacking the linker segment. In the context of T5, the linker region appears to be exposed on the outer portion of the molecule, eliciting strong immunogenic reaction and antibody production. Thus, while the linker segment is thought to be inert in the context of wild type heparanase, it appears as a functional entity in the context of the T5 splice variant.

Taken together, we provide evidence that T5 is a clinically relevant splice variant which may complement the pro-tumorigenic function of heparanase. T5 likely assumes a distinct three-dimensional folding other than that of heparanase, a notion that is now supported by our mAb 9c9. Expression of T5 in blood vessels and immune cells (Figs. 2C–E; 3D) may suggest that T5 function is not restricted to cancer but may well be involved in other pathological disorders (i.e., inflammation). Clearly, more research is required to resolve the crystal structure of T5 and to appreciate its contribution to the progression of other types of cancer and non-cancerous disorders. Studies aimed at these directions are ongoing.

## Supporting Information

Figure S1 **ELISA.** 96-well plate was coated with MBP (▪), MBP-T5 (▪), or heparanase (▪) proteins (1 µg/ml) and incubated with the indicated mAb for 2 h at room temperature. Following washes, anti-mouse IgG HRP-conjugated secondary antibody (Jackson Immunoresearch; West Grove PA) was applied and mAb binding was visualized by colorimetric reaction (TMB/H_2_SO_4_). Shown are representative OD values obtained.
(TIF)

Figure S2 **A**. **Schematic diagram of heparanase/T5 structure.** Heparanase is first synthesized as a pre-proenzyme, harboring 35 amino acids signal peptide (SP, Met^1^–Ala^35^) which is removed upon entering the ER. The protein is then subjected to glycosylation and secreted as a ∼65 kDa latent protein (upper panel). Proteolytic processing removes the linker domain (Ser^110^–Gln^157^), resulting in 8 kDa (Gln^36^–Glu^109^), and 50 kDa (Lys^158^–Ile^543^) protein subunits (second panel) that heterodimerize to yield an active enzyme. Replacement of the linker segment with three pairs of glycine (G)-serine (S) results in a constitutively-active single chain enzyme (GS3; third panel). The SP, 8 kDa and linker fragments are retained in T5, but the 50 kDa subunit is excised except for 9 amino acids, which are followed by the addition of three unique amino acids (SKK, lower panel). **B**. Epitope determination. HEK 293 cells were transfected with wild type 8 kDa gene construct or 8 kDa deleted at its C-terminus (Gln^36^–Ser^77^; 8ΔC) or N-terminus (Leu^65^–Glu^109^; 8ΔN). Control cells were transfected with an empty plasmid (Vo). Lysate samples were then subjected to immunoblotting applying mAb 5B5 (upper panels) or mAb 9D5 (second panels). Equal protein loading is exemplified by actin immunoblotting (fourth panel); Myc-tag immunoblotting confirms comparable expression levels of gene constructs (third panel). The epitope of both antibodies is localized at the protein N-terminus.
(TIF)

Table S1 **Performance of anti-T5 monoclonal antibodies.**
(TIF)
