# Supplementary figures and images for: Clinical Significance of Heparanase Splice Variant (T5) in Renal Cell Carcinoma: Evaluation by a Novel T5-Specific Monoclonal Antibody
Source: PLoS One. 2012 Dec 12;7(12):e51494. doi: 10.1371/journal.pone.0051494 (PMC3520799; doi:10.1371/journal.pone.0051494)

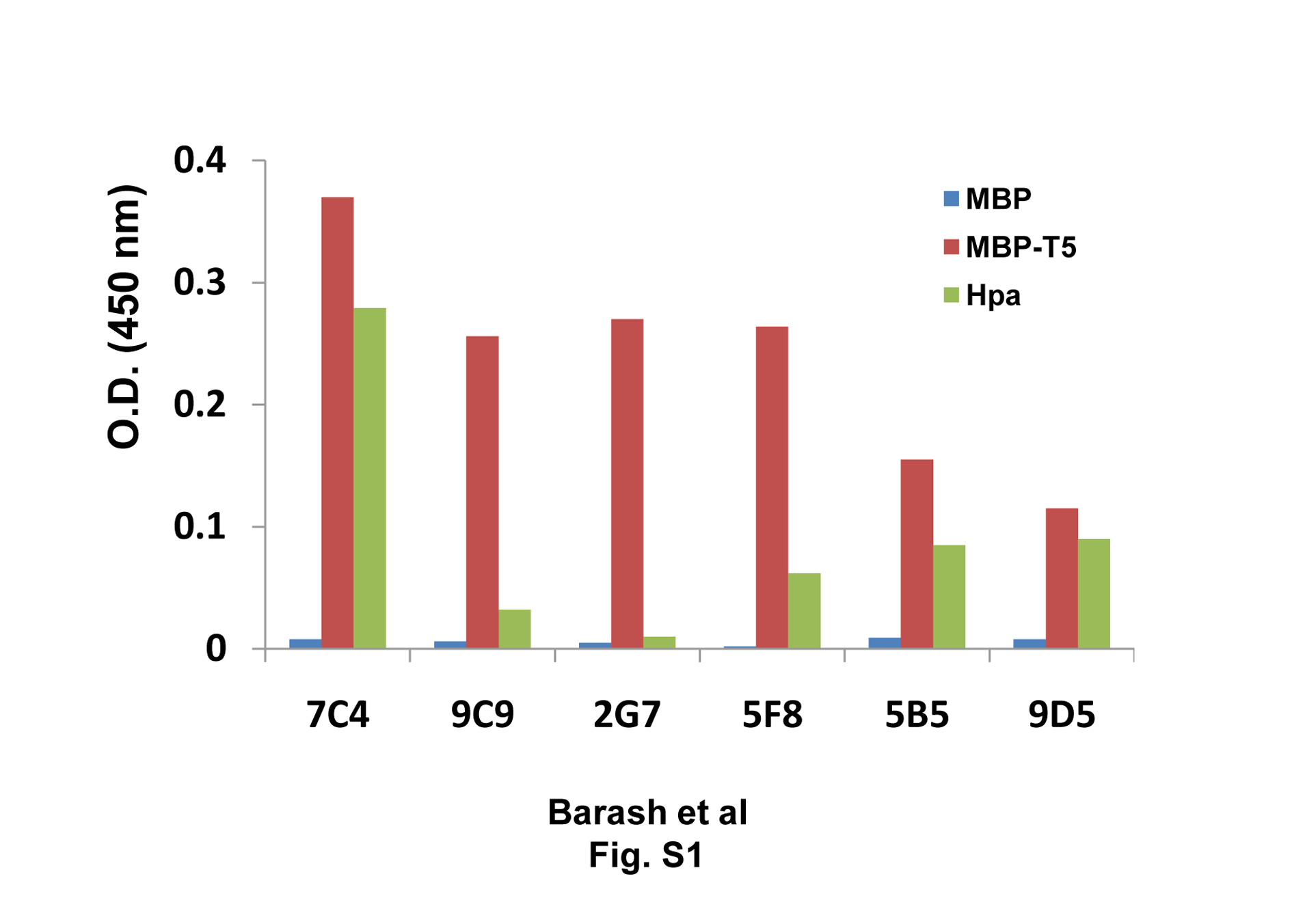

Supplement: Figure S1 — ELISA. 96-well plate was coated with MBP (▪), MBP-T5 (▪), or heparanase (▪) proteins (1 µg/ml) and incubated with the indicated mAb for 2 h at room temperature. Following washes, anti-mouse IgG HRP-conjugated secondary antibody (Jackson Immunoresearch; West Grove PA) was applied and mAb binding was visualized by colorimetric reaction (TMB/H2SO4). Shown are representative OD values obtained. (TIF) [file pone.0051494.s001.tif]

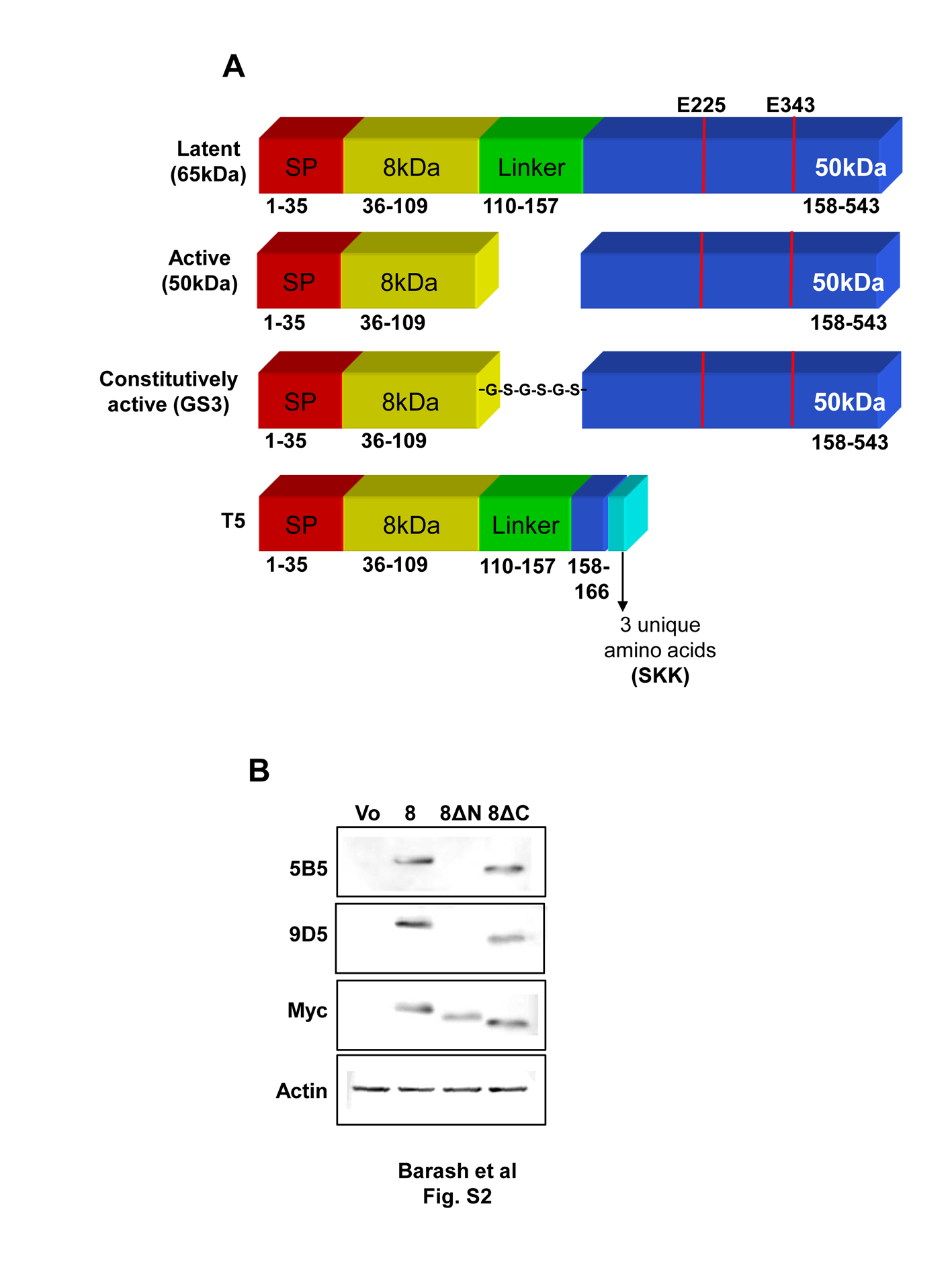

Supplement: Figure S2 — A. Schematic diagram of heparanase/T5 structure. Heparanase is first synthesized as a pre-proenzyme, harboring 35 amino acids signal peptide (SP, Met1–Ala35) which is removed upon entering the ER. The protein is then subjected to glycosylation and secreted as a ∼65 kDa latent protein (upper panel). Proteolytic processing removes the linker domain (Ser110–Gln157), resulting in 8 kDa (Gln36–Glu109), and 50 kDa (Lys158–Ile543) protein subunits (second panel) that heterodimerize to yield an active enzyme. Replacement of the linker segment with three pairs of glycine (G)-serine (S) results in a constitutively-active single chain enzyme (GS3; third panel). The SP, 8 kDa and linker fragments are retained in T5, but the 50 kDa subunit is excised except for 9 amino acids, which are followed by the addition of three unique amino acids (SKK, lower panel). B. Epitope determination. HEK 293 cells were transfected with wild type 8 kDa gene construct or 8 kDa deleted at its C-terminus (Gln36–Ser77; 8ΔC) or N-terminus (Leu65–Glu109; 8ΔN). Control cells were transfected with an empty plasmid (Vo). Lysate samples were then subjected to immunoblotting applying mAb 5B5 (upper panels) or mAb 9D5 (second panels). Equal protein loading is exemplified by actin immunoblotting (fourth panel); Myc-tag immunoblotting confirms comparable expression levels of gene constructs (third panel). The epitope of both antibodies is localized at the protein N-terminus. (TIF) [file pone.0051494.s002.tif]

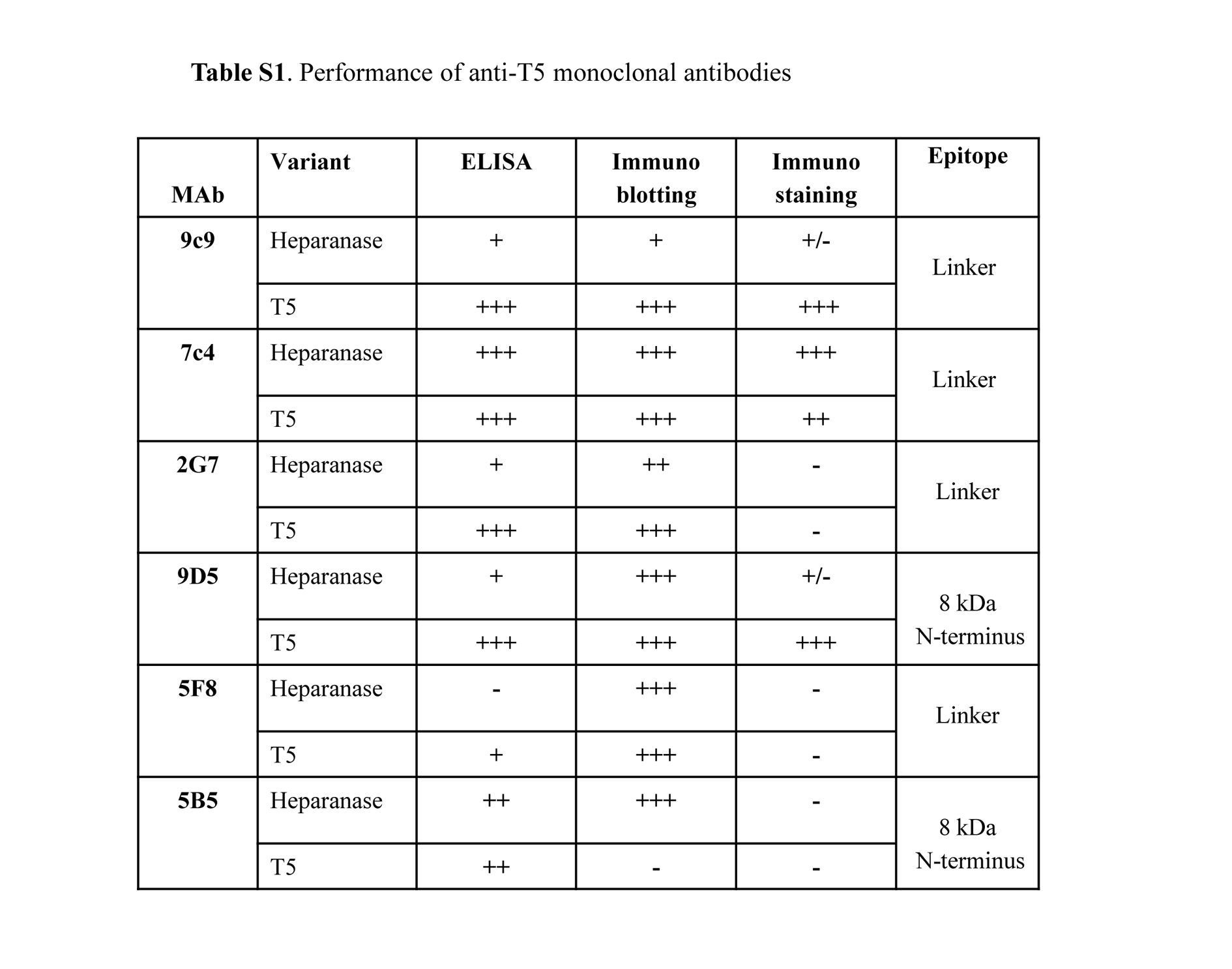

Supplement: Table S1 — Performance of anti-T5 monoclonal antibodies. (TIF) [file pone.0051494.s003.tif]
